# Supplementary material for: Novel Monomethoxy Poly(Ethylene Glycol) Modified Hydroxylated Tung Oil for Drug Delivery
Source: Polymers (Basel). 2023 Jan 21;15(3):564. doi: 10.3390/polym15030564 (PMC9921749; doi:10.3390/polym15030564)
Supplement: Supplementary file 1 [file polymers-15-00564-s001.zip › polymers-2152709-supplementary.pdf]

Supplementary Materials

Novel Monomethoxy Poly(Ethylene Glycol) Modified Hydroxylated Tung Oil for Drug Delivery

Huafen Wang [1,*], Huanhuan He [2], Jiaxiang Zhang [1], Juntao Liu [3], Yuwei Zhuang [1], Yuanyuan Yin [1], Zhiyong Ren [1], Yang Fu [1] and Suqin He [2]

[1] High & New Technology Research Center of Henan Academy of Sciences, No. 56 Hongzhuan Road, Zhengzhou 450002, China
[2] School of Materials Science and Engineering, Zhengzhou University, Zhengzhou 450001, China
[3] Faculty of Science, Henan University of Animal Husbandry and Economy, Zhengzhou 450001, China
* Correspondence: wanghuafen_2013@163.com; Tel: +86-371-65511668

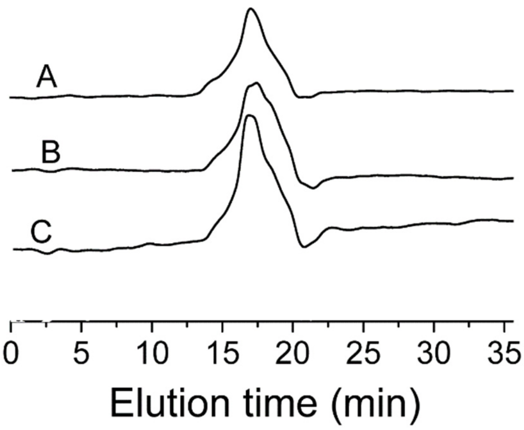

Figure S1. GPC curves of *m*PEG-HTO-*m*PEG amphiphilic polymers in THF: (A) *m*PEG-HTO-*m*PEG-1, (B) *m*PEG-HTO-*m*PEG-2 and (C) *m*PEG-HTO-*m*PEG-3.
